# Notes and News

**Published:** 1896-12-19

**Authors:** 


### Notes and News.

(Collected and Communicated.)

A rather serious outbreak of typhoid fever has occurred at Leeds. The medical officer of health has himself been attacked by the disease.

Professor Desgranges, late physician in chief to the Hotel Dieu Hospital in Paris, has bequeathed property valued at £20,000 to that institution, and £800 to the Rhone Association of Medical Men.

We are glad to learn that a sufficient sum has been subscribed by Dr. Cullingworth's medical friends to defray all the expenses connected with his recent legal action.

Scarlatina has been making its way in Dublin for more than a year, and the proportions it has assumed have compelled the authorities to realise the necessity of providing something towards adequate hospital accommodation for the patients. The Pigeon House Fort is to be utilised for those convalescing. The fort is not the property of the sanitary authorities, and it is reported the brunt of providing for the sufferers from the epidemic in the temporary hospital will fall upon the South Dublin Board of Guardians.

The offer of Dr. Barnardo and Mr. Quarrier to undertake the care of a large number of destitute Armenian children is creating quite an excitement. Dr. Barnardo offers to give a home to no less than 1,000 destitute Armenian orphan children, and Mr. Quarrier makes the same offer towards 100. Whether it will be possible to carry out this large scheme of benefiting alien children is not, of course, yet decided, but there is something very British in the idea which results in our extending the helping hand of brotherhood outside the large circle of our own ever-growing want. We do not recollect any foreign home being offered to the victims of the potato famine in Ireland, for instance, or of any other national calamity.

The new University at Clermont-Ferrand, in France, has been inaugurated.

The committee of the London Fever Hospital are appealing for funds to enable them to complete the reconstruction of the buildings at Islington and the establishment of a convalescent home, which they find is necessary to enable them to meet the demands made upon them.

At the Mercers' Hospital, Dublin, the governors have adopted the unusual course of requesting the present medical staff to resign at the end of the present year. Some of the members of the staff, it is expected, will be reinstated, as the resignation does not preclude from re-election.

A report that there are some cases of bubonic plague in London has created some sensation. Although the plague was reported to have reached Calcutta, the form of the epidemic was said to be very mild, and its existence was denied in some quarters. Now Calcutta is said to be free of the epidemic, whilst from Bombay we hear that there is no diminution.

In Germany the treatment of quacks is summary and effective. A Dusseldorf doctor, found guilty of selling nostrums for all kinds of disease and of advertising cures—all obvious impositions—has been sentenced to four years' imprisonment, a fine of £150 has been imposed, and his rights of citizenship have been suspended for five years.

It has been decided that Edinburgh is to have a new fever hospital. A suitable site, upwards of 350 feet above the sea-level, has been chosen and plans prepared. Separate accommodation will be provided for all manner of infectious diseases, and amongst these upwards of 300 beds are allotted to scarlet fever and ten to typhoid fever. The wards will be built on the pavilion system.

Charing Cross Hospital, we learn, is to benefit by one of Mr. Bancroft's admirable readings, which he has promised to give at St. Martin's Town Hall on January 22nd; and if we may judge from the audience which filled the Queen's Hall, when the reading was given on behalf of Middlesex Hospital, applications should be speedily made for tickets, or there will be many desirous of attending who will find themselves unable to gain admittance.

The constitution of the Leek Cottage Hospital is somewhat peculiar, in that it is governed under rules framed by the Charity Commissioners—rules which cannot be altered. On the archway leading to the hospital, however, there is an inscription stating that the hospital was given by Mrs. Alsop "to the inhabitants of Leek," and it seems that certain of the inhabitants have conceived the idea that "the inhabitants" ought to have the management of the whole affair in their own hands. According to the scheme of the Charity Commissioners, however, all the buildings and land are now vested in "the official trustees of charity lands," and all endowments have been transferred to the "official trustee of charitable funds," and the management of the institution is handed over to a committee elected by subscribers of five shillings and upwards. Clearly the inhabitants, as such, cannot interfere in the matter, but a five-shilling qualification surely ought not to keep anyone out who is likely to be of any use in the management of the hospital.

